# A Biopsychosocial Overview of the Opioid Crisis: Considering Nutrition and Gastrointestinal Health

**DOI:** 10.3389/fpubh.2019.00193

**Published:** 2019-07-09

**Authors:** David A. Wiss

**Affiliations:** Fielding School of Public Health, University of California, Los Angeles, Los Angeles, CA, United States

**Keywords:** biopsychosocial (BPS) model multidisciplinary, opioid, nutrition, gastrointestinal, trauma, addiction, substance use disorder, microbiome

## Abstract

The opioid crisis has reached epidemic proportions in the United States with rising overdose death rates. Identifying the underlying factors that contribute to addiction vulnerability may lead to more effective prevention strategies. Supply side environmental factors are a major contributing component. Psychosocial factors such as stress, trauma, and adverse childhood experiences have been linked to emotional pain leading to self-medication. Genetic and epigenetic factors associated with brain reward pathways and impulsivity are known predictors of addiction vulnerability. This review attempts to present a biopsychosocial approach that connects various social and biological theories related to the addiction crisis. The emerging role of nutrition therapy with an emphasis on gastrointestinal health in the treatment of opioid use disorder is presented. The biopsychosocial model integrates concepts from several disciplines, emphasizing multicausality rather than a reductionist approach. Potential solutions at multiple levels are presented, considering individual as well as population health. This single cohesive framework is based on the interdependency of the entire system, identifying risk and protective factors that may influence substance-seeking behavior. Nutrition should be included as one facet of a multidisciplinary approach toward improved recovery outcomes. Cross-disciplinary collaborative efforts, new ideas, and fiscal resources will be critical to address the epidemic.

## Introduction

The opioid crisis in the US has received extensive coverage in the media leading to increased awareness of this “public health emergency.” Between 2000 and 2014, nearly a half million people in the US died from a drug overdose (1). Opioids accounted for 61% of all drug-related overdoses in 2014 (1). Overdose death rates are highest when opioids and benzodiazepines are combined (2). It has become increasingly clear that over-prescription of these medications in the past two decades is a primary upstream driver of the crisis. The rapid rise in costs associated with addiction treatment threatens the infrastructure and finances of many US hospitals (3). According to some estimates, the number of people currently dependent on opioids or heroin is more than three times greater than the current capacity to deliver treatment (4). The President's Fiscal Year 2017 budget proposed allocating a billion dollars in an effort to reduce prescription drug misuse through the twenty-first Century Cures Act. However, despite increased resource allocation, policy changes, and changing cultural norms about addiction, little measurable progress has been made in reducing the problem. Relapse rates continue to be above 50% at 6 months (5, 6) and similarly high worldwide (7, 8).

Given the current crisis and the alarming rates of overdose and death associated with prescription opioids, illicit synthetic versions (i.e., carfentanil), and street heroin (1, 9), researchers have focused on identifying factors that contribute to addiction vulnerability (10). Numerous authors have hypothesized that there is an interaction between genetic factors (innate predisposition) and environmental and personal factors (collectively referred to as psychosocial factors herein). Furthermore, efforts to effectively reduce the opioid epidemic will require understanding individual differences that contribute to drug use initiation, as well as long-term neurobiological adaptations stemming from prolonged intake. Knowledge of these interactions may lead to improved treatment protocols that account for underlying vulnerability. Because the opioid problem is heterogenous, a wide range of treatments will be needed to target various geographical regions, age groups, and addiction severity. This review attempts to explore the interplay between social and biological factors, including potential mediators related to opioid use. A strong case will be made to consider nutrition in the treatment of opioid use disorder (OUD), whereas the role of opioid maintenance therapies is emphasized less due to extensive coverage elsewhere (11, 12).

### Pain Management

In 2001 The Joint Commission identified self-reported pain as a fifth “vital sign” for healthcare providers to consider, which led to the liberal use of pain-relieving medications. It has been suggested that the opioid crisis may be seen as a dual epidemic: one of abuse, and the other as the right to control poorly defined pain (13). While the concept of “self-reported pain” has generated considerable debate, the increasingly negative consequences associated with analgesics (pain killers) have necessitated intervention at multiple levels including hospital emergency departments where misuse and diversion are common (14). There is an urgent need for health care professionals to educate and realign patient expectations regarding pain management (15). By far the most common acute and chronic pain medications are opioid analgesics which include codeine, hydrocodone, oxycodone, morphine, and fentanyl, and others. In a study using data from 2000 to 2005, over half of patients taking prescription opioids beyond 12 continuous weeks were still using them after 5 years (16) underscoring the addictive potential of these drugs. Between 2002 and 2014 the odds of young adults (ages 18–34 years) having a prescription OUD doubled (17). In response to escalating abuse, makers of OxyContin released an “abuse-deterrent formula” in 2010 at which time heroin use began to rise (18). Between 2010 and 2014 heroin-related deaths tripled in the US (1).

### Medication Assisted Treatment

The Comprehensive Addiction and Recovery Act (CARA) signed by President Obama in July 2016 expanded funding for the availability of medication assisted treatment (MAT) for OUD. MAT consists of pharmacotherapy, ideally in conjunction with behavioral health intervention. Medications such as methadone and buprenorphine (Subutex) have proven effective in mitigating the negative side effects associated with OUD (19). More recently Suboxone (buprenorphine plus naloxone) has replaced Subutex due to its lower abuse potential. Naloxone (Narcan) can reverse the effect of overdose and is recognized by CARA as a primary agent for saving lives. Naloxone is a competitive inhibitor of brain opioid receptors, while naltrexone is a similar blocking agent used for relapse prevention by impeding the euphoric effect of opioids, as well as the rewarding effects of alcohol. The use of MAT has grown in recent years and is now considered the most common practice for treating OUD. MAT can be considered a “harm reduction” or “risk mitigation” strategy compared to traditional models of addiction treatment which have focused on complete abstinence after the detoxification period. Ideally, the goal of MAT is to move patients toward abstinence, but many stay on MAT for extended periods of time.

### Theoretical Framework

The biopsychosocial (BPS) framework was originally proposed in 1980 by Dr. George Engel stemming from his dissatisfaction with the biomedical model of illness (20). BPS draws its conceptual roots from the general systems theory which originated in the 1950s and aimed to unify knowledge and theories across different disciplines into a systematic vision of a “better world” (21). The BPS model has been promoted by the field of psychosomatic medicine utilizing “mind-body” approaches to health (22) that are common in “alternative therapies” (e.g., meditation, acupuncture, nutrition). A central theme with this approach is the use of seemingly divergent conceptual models to emphasize multicausality in understanding disease, rather than a reductionist approach. An example of a similar approach known as Ecosocial Theory was introduced by Krieger (23). The interactive BPS model proposes an integrated vision of health and disease that does not focus on a single root cause which is seen in a traditional biomedical approach (24). The integration of social and biological processes (25) may be critical for OUD treatment since the reductionist biological model has not been productive (and arguably harmful) and capitalized on by the pharmaceutical industry (26). A BPS framework not only helps guide addiction treatment, but also influences public perception of addiction (27).

**Figure 1:**
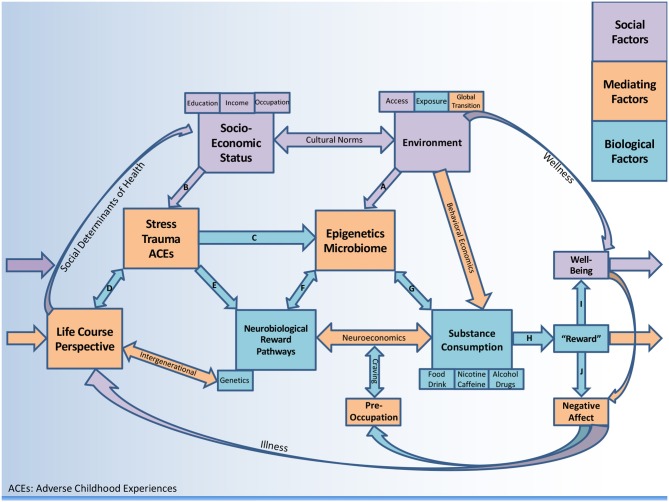
A biopsychosocial perspective on substance consumption.

This comprehensive review examines the opioid crisis using a biopsychosocial framework (see Figure 1) with particular emphasis on (1) social and environmental factors (2) psychosocial factors (stress, trauma/adversity) and (3) biological factors (including potential mediating mechanisms). In analyzing the opioid crisis at the individual as well as population level, a case will be made for considering alternative treatment modalities for OUD such as the emerging role of nutrition, with emphasis on gastrointestinal (GI) health.

## Social and Environmental Factors

### Environmental Factors

Many researchers and health care professionals believe that the opioid epidemic is mostly a consequence of “supply side” abundance, resulting from aggressive marketing by the pharmaceutical industry as well as physicians who have over-prescribed. A recent public opinion poll identified physicians as being responsible for the crisis (28). Given the link between prescription opioid use and later onset of heroin abuse, an obvious public health strategy is to focus on reducing improper opioid prescriptions. The Center for Disease Control (CDC) has recently issued guidelines stating that non-opioid therapy is preferred for chronic pain outside of active cancer, palliative, and end-of-life care (29). Other authors believe that restricting the ability of physicians to write prescriptions is only a short-term fix (30). New Cures Act requirements for prescribers are currently underway.

Supply-reduction efforts have had some success (18). However, evidence suggests that those already dependent on prescription opioids frequently transition to heroin when their supplies are cut off (31). Data from 2015 shows that a third of people in treatment for OUD began with common prescribed opioids and progressed to heroin use (31). Other research has shown that as many as 80% of heroin users started with prescription opioids (32). There is a genuine concern that individuals who originally benefited from opioid medications will turn to purchasing drugs from an illicit market if their prescriptions are stopped (33). Recent data shows alarming rates of chronic pain preceding the onset of OUD, associated with high rates of mental disorders (81.7%), suggesting a high risk of transitioning to illicit drugs if stopped abruptly (34).

Can the issue of opioid misuse be tackled on the supply side alone? History suggests not. Legislative efforts at the State level to close “pill mills” have had little discernable impact in reducing opioid use (35). Other recommendations to tighten control include protocols to ensure authenticity of the prescription source, adding additional abuse detection steps, and practices for returning unused drugs (36), as well as more physician education during residency training (37). While it is hopeful that policy interventions can reduce overdose and death, solutions to control illegal heroin coming into the country are less obvious and fall under the jurisdiction of the Drug Enforcement Agency (DEA). Heroin arrests have been increasing in some states (38). Interventions focusing only on prescription opioids are unlikely to be sufficient as long as heroin and other synthetic opioids such as fentanyl and carfentanil continue to flood the market. Illicit drugs are increasingly available through the “dark web” and are an important but less documented mechanism driving the opioid crisis.

The environment in which one resides is a known predictor of consumption behavior (39), thus behavioral economics is a conceptual system to understand how one's access and exposure will predict demand and subsequent intake (40–43). Historically, research on human choice has been dominated by economic theory. Eventually it became clear that the quest to “maximize utility” could not capture human preference (44) nor would it apply to disorders such as addiction. Behavioral economics is a scientific discipline at the intersection of economics and psychology as it pertains to health-related behavior (45). It has been used to study decision making in the context of substance use disorders (SUD) (46–48) including alcohol (49) and other health behaviors such as food and drink consumption (40, 50). This construct may be useful to make connections between the environment and consumption behavior (Figure 1).

### Socioeconomic Status (SES)

The relationship between opioid prescribing practices and SES has not been extensively studied. US data from 2006 to 2009 suggests that patients presenting to emergency departments from lower SES regions were less likely to receive opioids for equivalent levels of pain compared to those from more affluent neighborhoods (39). In a more recent study of the association between new back pain diagnosis and opioid medication use, low neighborhood SES has been linked to significantly higher opioid prescription rates, suggesting the possibility of higher inappropriate narcotic use (poor physician guideline compliance) in less advantaged areas (51). Meanwhile, heroin use has significantly increased across most demographic groups (41). In an ecological study of one Southern California county from 2010 to 2014 (*n* = 1,205) higher education and income were protective against opioid-related deaths but the data suggests that no group is immune and there is dire need for public health interventions at all SES levels (42).

Norwegian data suggests that individuals with a drug-related death had lower SES than the general population but overall SES situation prior to death was heterogenous (41). A large dataset from Kaiser Permanente Northern California showed that prescription opioid use is lowest in the most deprived neighborhoods (52). Conversely, other Kaiser reports using a larger dataset have found individuals living in deprived neighborhoods are more likely to become long-term users (53). The CDC has examined county-level factors associated with prescribing patterns and have found higher opioid use in regions with higher rates of unemployment and Medicaid enrollment (54). Pregnant women using opioids as their primary substance have the highest prevalence in the Southern US and are less educated (43). Other research on pregnant women in the US have shown similar education gradients and higher opioid use below $20,000 annual household income (55) which is consistent with national data linking lower levels of income to opioid misuse (56).

In a rural part of Wisconsin (92.6% White, median income $46,333), a majority of overdose patients had private insurance (57) which is contrary to national data suggesting higher rates of overdose among the uninsured (41). Given that SES is an important predictor of health care utilization, more research is needed on OUD in uninsured populations in order to truly capture the effect of SES on opioid prevalence rates and health outcomes. Recent data suggests opioid use plays a critical role in fueling rising suicide rates (58). Inconsistent findings between SES and opioid misuse and death necessitates longitudinal data in order to track changes in these relationships over time.

Despite these inconsistencies in the opioid literature, in public health SES should always be considered when examining health outcomes and building interventions. The BPS Perspective considers how socioeconomic disadvantage can be deleterious (Figure 1, path B), or how socioeconomic advantage can be protective against negative outcomes associated with drug use (via environments that support wellness). Figure 1 includes a pathway where substance consumption stimulates “reward” (discussed in section Reward Pathways) leading to “well-being” (path I) which does not feedback to negative affect and craving. The model considers multiple substances (including food, beverages, and caffeine) which are generally less subtle (less dopaminergic) in their addictive potential when compared to drugs, alcohol, and nicotine. Thus, the framework is designed to conceptualize negative health outcomes but is flexible regarding psychosocial protective factors, therefore comprehensive and not limited to OUD.

## Psychosocial Factors

### Trauma and Stress

Efforts to reduce supply alone are unlikely to resolve the opioid abuse problem in the US. It is possible that some of the pain associated with opioid dependence is psychological. In other words, opioid misuse may be a coping mechanism for unresolved emotional pain that cannot be easily addressed in other ways. Allostasis describes the body's adaptations to predictable or unpredictable changes in the environment. McEwen's concept of allostatic load (59) is an early example of how social and biological factors integrate to influence health outcomes. More recently Koob fit an allostatic model to addiction where the brain is challenged to self-regulate under stress, and subsequent changes in corticotropin-releasing factor (CRF) further compromise neurocircuitry (42).

Post-traumatic stress disorder (PTSD) causes changes in fear and stress-related biology including hyperarousal, trauma cue-dependent recall, avoidance, and extinction memory deficits, among others (60). According to one study, numbness or detachment as a result of trauma exposure appears to be the PTSD symptom most strongly associated with pain-related outcomes (61). Co-occurring PTSD and SUD has also been associated with insomnia (62) which can negatively affect health. A recent study on veterans demonstrated that buprenorphine use was associated with a significant improvement in PTSD symptoms after 8 months (63) highlighting overlapping mechanisms between SUD and PTSD.

Neuroimaging studies have shown that trauma has a measurable, enduring effect on the functional dynamics of the brain, even in the absence of clinically diagnosable PTSD (64). In a large national sample, the presence of PTSD increased the risk of developing OUD after exposure to opioid painkillers (65). These findings suggest that neurobiological imprints of PTSD such as the release of CRF during periods of activation/arousal increase susceptibility to addiction. Recent evidence suggests that the association between PTSD and opioid use is more pronounced in women than men (66). A recent case study described a woman who was diagnosed with major depressive disorder and OUD, but later was identified as using opioids to self-medicate her underlying undiagnosed PTSD (67).

Deficits in reward functioning may be a mechanism underlying anhedonia (lack of pleasure) associated with PTSD (68). The trauma theory suggests that opioids are distinctly reinforcing to individuals with PTSD (69). While this explanation will not occur in all cases of OUD, it may represent a distinct subtype. It appears likely that physical changes associated with trauma create increased risk for SUD, lending support to the BPS Perspective (path E). Better detection and integrated treatment for comorbid PTSD and OUD may be helpful. Several authors have suggested that PTSD screening should be routine for clinicians who prescribe opioids (65), particularly for chronic pain syndromes (61).

### Adverse Childhood Experiences

A considerable amount of research has connected adverse childhood experiences (ACEs) to a dose-dependent increase in risk for drug abuse (70, 71). Strong links between ACEs and the initiation of opioid use have been described (72, 73). ACEs have been linked to age of opioid initiation, intravenous use of the drug, and lifetime overdose in a graded, dose-response manner (73). Potential mechanisms mediating this relationship could be environmental (e.g., poverty, parental criminal justice involvement) as well as biological (e.g., genetic heritability, altered neurodevelopment). Given the significant associations with childhood abuse and prescription opioid use, several authors have identified child maltreatment as an important social and environmental factor (path B) which should be considered in prevention and intervention efforts amidst the crisis (74, 75). Some authors have suggested that resources should be invested into policies and programs that prevent ACEs as a mechanism to reduce substance misuse (76). Overall, the findings underscore the importance of OUD treatment being guided by trauma-informed modalities, including “complex trauma” (different from PTSD) (77).

### Psychosocial Vulnerability

The “brain disease model of addiction” has been challenged by some authors who advocate for an addiction disease model that includes the presence of a pre-existing disorders such as anxiety or depression (78). Rodent studies have demonstrated that social isolation leads to an increase in drug self-administration (79). Sociological research has identified that neighborhoods with high crime and deviance rates are associated with higher rates of opioid misuse among adolescents ages 12–17 living in socially disorganized areas (80). Substandard environmental factors related to SES and structural racism can “get under the skin” and create health problems including addiction (81). The social determinants of health are thus an important part of the overall BPS Perspective on opioids, linking the Life Course Perspective (see section Life Course Perspective) to SES and environment.

The PTSD susceptibility model suggests that OUD can lead to increased psychosocial vulnerability via negative experiences associated with drug using (procurement, intoxication, increased risk of accidents, and violence) (69). However, the more common theory is the “self-medication hypothesis” (69, 82) which suggests individuals turn to opioids to reduce stress, pain, and unresolved psychological trauma (path C–F). This hypothesis has also been described as “latent vulnerability,” suggesting that childhood neglect increases the lifetime risk of developing a psychiatric disorder (83). One proposed mechanism of vulnerability to addiction includes a compromised ability to regulate emotions effectively (84). Other research has identified increased impulsivity as a significant moderator between PTSD and substance misuse (85, 86) as well as the role of negative urgency within this relationship (87). More research is needed on the direct link between stress and dopaminergic reward pathways associated with OUD.

## Biological Factors

### Reward Pathways

Consumption of substances activate mesolimbic reward pathways (path H). Despite some disagreement (88), most authors understand SUD to be a brain disease associated with weakened executive functioning leading to poor self-regulation and repeated relapse (89, 90). Neuroimaging studies have revealed biomarkers in the corticolimbic (91) and corticostriatal regions that may be predictors of relapse (92) in the face of drug cues (93). Altered neurotransmission in frontostriatal circuits have linked multiple forms of impulsivity to drug-seeking behaviors (94). A specific understanding of the reward process related to the opioid epidemic necessitates an understanding of pain pathways viewed as anti-reward processes associated with dopamine (DA) deficits (95). A recent review describes how inflammatory processes may decrease DA synthesis and availability via multiple pathways (96).

Animals models have demonstrated impaired incentive learning in early opioid withdrawal resulting in maladaptive reward seeking (97) which in some cases can last a lifetime (78). The persistence of a learned association with pain relief provides the continued motivation for seeking opioids, particularly in the face of distress or dysphoria (path E). These learned associations of relief from an aversive mental state, either pre-existing or created by the withdrawal drives the craving cascade in susceptible individuals (78). It is likely that repeated use perpetuates anhedonia, and thus interferes with chances of long-term recovery (98). Negative affective states during the period after substance consumption are an important part of the withdrawal-craving cascade (path J). It has been shown that in the absence of the substance, negative moods (e.g., depression, anxiety) coupled with enhanced sensitivity to stress eventually create obsession-like preoccupation (brain becomes “hi-jacked”), a loss of executive functioning, and then relapse, reinitiating the cycle again (89). In Figure 1 the preoccupation-craving feedback loop converges with the neuroeconomics construct (see section Neuroeconomics). It is also worth acknowledging that “well-being” or euphoria typically precedes dysphoria (negative affect) and is the basis for incentive salience that generally motivates the entire cascade (42). People without biological and psychosocial vulnerability who have not been overexposed can experience the perceived positive effects of dopaminergic substances without developing an addiction (path I).

Taken together, neurobiological drivers of OUD should be considered in the context of the current epidemic, and potential solutions ought to look beyond pharmacology alone. It is unknown how a nutrition intervention might modify reward pathways over extended periods of time (i.e., years). Given the emerging data on food addiction (99, 100), it is believed that reducing exposure to highly palatable foods may have a noticeable neurochemical impact when assessed over the lifespan (albeit very difficult to measure in humans). Given the neurochemical overlap between food and drugs of abuse, it is not implausible to anticipate changes in behavior (e.g., sobriety from drugs) via alterations in other consumption behavior. At a minimum, nutrition interventions may improve the body's resilience in response to stress and negative affect throughout the recovery process, but this is unproven.

### Genetic Vulnerability

Genetic research has identified polymorphisms in dopaminergic genes and other neurotransmitter variants which may put individuals at an increased risk of impulsive behavior and addiction (94). The heritability of impulsivity has been linked to a range of genes known as DAT, MAOA, and COMT (101) suggesting that no single gene can predict impulsivity in humans. More recent data points to loci within the HTR2A gene (encodes a serotonin receptor), casting some doubt on the previously identified candidate loci for impulsive personality traits (102). Importantly, with elevated stress levels there appears to be a cumulative effect on vulnerability to OUD (103). The concept of reward deficiency syndrome (RDS), introduced by Blum et al. (104), identified the dopamine D2 receptor (assessed by A1 allele) as the primary site for substance-seeking behavior. Interestingly, DAD2 dysfunction has also shown associations with increased risk of PTSD (105). Blum and colleagues created the Genetic Addiction Risk Score (GARS) as a marker for predisposition to RDS (106). It is rare that a single gene predicts behavior (44) and to date there is no convincing data that any one gene can transmit addiction to future generations (107). While there are several possible genetic markers, DAD2 receptor dysfunction has shown the strongest association with addiction vulnerability but it remains unclear if low DAD2 is genetically determined, or merely a consequence or prolonged drug abuse. Meanwhile, multiple lines of study have linked distinct subtypes of impulsivity and risk-related decision making to low DAD2 receptor function (94). DA has been referred to as the “anti-stress molecule” and receptor dysfunction may drive substance-seeking behavior under distress and is an important component of the BPS Perspective (path E, and path C–G).

### Neuroeconomics

Neuroeconomics is behavioral economics plus neuroscience (108) and has been referred to as “decision neuroscience” (109). Techniques such as functional magnetic resonance imaging (fMRI) have introduced biophysical data into behavioral economics in order to understand how value maximization is computed at the neural level. These include choice anomalies (44), deviation from rationality (49), and delay discounting (preferences for smaller immediate rewards relative to larger delayed rewards) (49, 110). This emerging field is highly relevant to our current understanding of SUD as a neurobiological disorder which impairs information processing (111). Several circuits responsible for processing input can lead to craving and relapse, including disorders of storage (learning and memory) and disorders of access (to decision making processes) (111). Executive dysfunction has also been associated with stress and has been linked to the SES gradient, as well as negative health behaviors (112). Neurobiology should play a more central role in our theoretical understanding of valuation (110) and choice (44). Efforts to understand circuit-specific variation in different individuals has the potential to tailor disease-augmenting therapies. Neuroeconomics can be viewed as a mediator between reward pathways and consumption behavior in Figure 1, with more details reviewed elsewhere (108, 113).

## Biological Mediators

Interest in the biological imprint of trauma has been growing. The biological correlates of complex trauma have been described across various brain regions (e.g., hippocampus, amygdala), throughout the autonomic nervous system (e.g., vagus nerve), in various neurobiochemical measures (e.g., cortisol), and across genetic as well as epigenetic factors (41) (path C). Environmental stress has the potential to alter lifelong hypothalamic-pituitary-adrenal (HPA) axis function and to induce subsequent neurodevelopmental maladaptation (51) (path A). The HPA axis is important for the production of glucocorticoids (e.g., cortisol) in response to physical and mental stress. Research elucidating mechanisms which link social and environmental factors to individual physiology is still in its nascent stages.

### Epigenetics

Epigenetics describes the interaction of genes with their environment. Maternal child health research has identified “critical periods” where epigenetic modifications are particularly impactful (51). This emerging field looks at changes that occur in the brain as a result of drug administration, with particular interest at mu-opioid receptor cites in the nucleus accumbens and ventral tegmental area (114, 115). Epigenetic changes during stressful social circumstances may predispose individuals to drug abuse (116) (path F). *In utero* stress exposure has been associated with DNA methylation changes leading to long term alterations in gene expression (117) which can alter the course of brain development (118) (path D). Prenatal exposure to maternal stress has been associated with a range of mental health disorders (119) including the development of eating disorders (120). Animal models have demonstrated that addiction-like eating during gestation and lactation can program the offspring for addiction-like behaviors including drug-seeking (121, 122). Given the overlap between nutritional programming and altered incentive motivation via the mesolimbic dopaminergic system (123), it is being hypothesized that eating behaviors in recovery from OUD may impact reward pathways and be mediated by epigenetic and microbial mechanisms. Further study on the impact of nutrition on genetic expression of addiction traits are warranted.

### Microbiome

Research on the gut microbiome has increased exponentially in the past decade. A state of equilibrium (i.e., homeostasis) serves health, whereas a compromised state (e.g., gut permeability) promotes dysbiosis, inflammation, and susceptibility to disease (124). Many authors have suggested that our gastrointestinal microbiome may be a key factor impacting our emotional and behavioral health (125). Changes in the composition of gut microbial profile (including byproducts from degradation of food) have been shown to modify regulation of genes (epigenetics) involved in depressive disorders (126). Systematic reviews have suggested positive effects of probiotics on depressive symptoms (127, 128). Diets rich in fiber and omega-3 have been shown to reduce the risk of depression, anxiety, and stress (129). Investigators have begun to consider potential connections between PTSD and the microbiome, mediated by the immune system and HPA axis (130) (path C). This area of investigation is an excellent example of how environmental, psychosocial, and biological factors clearly interact to influence health. The microbiome as a mediator has created a paradigm shift in neuroscience and psychiatry (131), highlighting the importance of nutrition that goes beyond the basics of macro- and micro-nutrients. A recent publication states: “attention to the microbiome may help answer nagging questions about the underlying biological mechanisms that link social conditions to health” (132). Some social scientists may view this as a reductionist approach, but it may prove to be the opposite.

The role of the gut-brain axis in determining food reward (133) has led to the possibility that microbes inside our intestinal tract may be influencing our consumption patterns (134, 135) (path G) through conditioned food preference via hormonal and dopaminergic mediators (133) (path F). It has been argued that bacterial species aim to increase their chance of survival (just like other organisms) and have a wide range of mechanisms to influence host consumption behavior, including production of neurotransmitters and short-chain fatty acids, manipulation of intestinal barrier function, and signaling along the vagus nerve (134, 136). This “puppeteer” theory has been challenged by the argument that microbial ecology has local effects on the gut stemming from an evolved dependence rather than direct human behavioral manipulation (137). Meanwhile, authors have speculated on the possibility that alterations in the gut ecosystem may be part of the etiology and progression of eating disorders (138).

The question is: how is the microbiome linked to AUD or SUD? This emerging topic considers neuroendocrine pathways that are involved in addiction, where gut microbiota may play a causal role (139). In regard to alcohol, several lines of evidence in both animals and humans have demonstrated a gut-liver axis which links inflammation, intestinal permeability, and immune function, to both liver and colon disease (140–143). More recently it has been proposed that dysbiosis associated with alcoholism induces neuro-inflammation via the central nervous system which can produce anxiety, depression, craving, as well as drinking behavior (144, 145). Links between the microbiome and OUD will be described in more detail below.

## Opioids and Nutrition

There is a paucity of high-quality evidence regarding the role of nutrition in OUD recovery. Nutrition does not easily lend itself to randomized controlled trials given the amount of time needed for measurable outcomes, and the presence of confounders introduced during this period. Therefore, nutrition research has been constrained to reductionistic approaches, such as looking at single nutrients or single outcome measures such as changes in weight. Conducting research on SUD populations creates additional challenges, as there are often high attrition rates (146). Biopsychosocial approaches to future nutrition research will hopefully renegotiate the boundaries between physical and mental health by targeting the gut-brain axis and examining novel outcomes.

### Eating Behavior

A high preference for sugar and sweetened foods has been consistently described during early abstinence from opioids (147–149). Not surprisingly, available evidence suggests low fiber intakes (147, 150). During active heroin use, individuals report little interest in food, preferring quick and cheap convenience foods (151). During early abstinence there is evidence of binge eating and addiction-like eating behaviors (152) as well as concerns about weight gain (153). Individuals on methadone maintenance predictably gain weight (154) particularly among females (155), and this effect is higher for individuals with less knowledge about healthful eating (156). It is possible that altered hormones associated with heroin addiction contribute to abnormal weight changes (157) and/or decreased bone mass (158). Less is known about how eating behaviors during the early months/years of recovery impacts reward processing. Given that highly palatable food can be very rewarding, it is not surprising individuals in early sobriety seek out these foods (159). A study evaluating the impact of nutrition on the reward-related neurochemistry of OUD patients has not been done.

### Nutrient Deficiencies

Vitamin and mineral deficiencies associated with opioid addiction have been well-described (160–165). However, given the retrospective nature of the research, it is difficult to determine if deficiencies are caused by poor dietary habits, by the drugs themselves, or possibly from impaired absorption. Opioid users can be considered at high nutritional risk (166) based on self-reported nutritional intake (167). Additionally, most of the opioid research has been conducted during methadone maintenance (162, 168–170), due to the difficulties conducting research on individuals using illicit drugs. Some studies have shown elevated serum values of malondialdehyde (167), homocysteine (171), and leucocytes (171), all of which serve as markers of inflammation. To date, a study on the use of nutrition therapy to reduce inflammation in OUD subjects has not been conducted.

### Gastrointestinal Health

OUD has been associated with bowel dysfunction including but not limited to constipation while using and diarrhea during detoxification (172). While laxatives and other over-the-counter remedies can be used to treat opioid-induced bowel dysfunction, they do not address underlying causes which may include microbial alterations (173). Several papers have addressed opioid-related GI concerns, yet no authors make any specific nutrition recommendations (174, 175). Given how challenging it is to conduct nutrition interventions in SUD detox settings, it is not surprising there is a lack of evidence. Meanwhile, clinical anecdote suggests that attention to a well-balanced diet during detoxification can minimize the intensity and duration of rebound diarrhea. Clinicians have been relying on case reports rather than published standards.

Activation of mu-opioid receptors in the gut wall inhibits pathways within the enteric nervous system, which in turn reduce motility, delay gastric emptying, and slow intestinal transit (172). Constipation typically persists as long as opiates are being administered, and emerging microbiome data presents compelling new questions related to the origins and consequences of pathophysiological motility. It has been shown that GI transit times are prolonged in the cecum and ascending colon, but not in the transverse or descending colon (175). Delayed gastric emptying (176) may create a motionless environment favorable to bacterial growth (177). It is possible that delayed GI transmit time can increase intraluminal concentrations of toxins. Chronic opioid use in cirrhosis has been associated with increased endotoxemia, gut dysbiosis, inflammation, and all-cause hospital readmission (178).

Recent findings indicate that gut microbiota modulates physiological responses related to tolerance induced by chronic morphine administration (179). In a rodent model using morphine, a particular strain of bacteria (*E. faecalis*) increased 100-fold compared to placebo (180). These findings have been replicated, and it has been added that certain microbial communities associated with stress tolerance are reduced in the morphine-rodent model (181). Similar to alcohol, opioid ingestion can disrupt the intestinal epithelium (160) leading to bacterial translocation and subsequent inflammatory cascades (161). Animal models that have opioid-induced gut microbial disruption, altered cholesterol/bile acid metabolism and systemic inflammation can be “rescued” by microbiota fecal transplantation positively influencing gut health (162). It is not yet clear whether the microbiome can contribute to craving in OUD, or to what extent opioid-induced dysbiosis impacts mental status in humans. However, it has been shown that antibiotic-treated rodents transplanted with saline microbiota have restored reward functioning (163). Rodent models have also shown that gut microbiota plays a key role in pain (166). Gut microbiome alterations and impulsive behaviors influenced by striatal dopamine receptor expression have reduced alcohol seeking in animal models (164). Meanwhile, interest in reducing neuroinflammation in opioid recovery is beginning to receive considerable attention (165). The gut-brain axis appears ripe for intervention strategies in OUD.

Given what is known about the links between gut and brain, it can be hypothesized that the microbiome is an important and modifiable mediator of substance-seeking behavior (paths F and G). It has been proposed that gut bacteria can influence neurobehavior including host appetite for food (135, 137, 182), so it is not implausible to predict associations between microbiota and all substances passing through the gut. A recent review summarizes bidirectional associations between drugs and bugs concludes: “it is not bizarre to think that in the future microbiome measures will form part of clinical practice to investigate either the efficacy or side effects of psychotropic compounds” (183).

## Discussion

### Theory Comparison

Efforts to address the opioid epidemic are being led by pharmaceutical companies promoting new medications (MAT) as the solution. One major shortcoming is that it does not address individuals' underlying psychological and emotional issues that contribute to addiction susceptibility. It examines the “macro” but not the “micro” environment, and one could argue that psychosocial factors require increased public health attention.

The psychosocial theory of addiction vulnerability is focused on the individual but is highly dependent upon social and environmental factors (path B). Disparities in population health are known to differ on the basis of social rather than biological factors (168). Individuals with a history of PTSD, complex trauma, stress, or ACEs can experience physiological as well as emotional changes that increase the likelihood of opioid addiction. The trauma theory of addiction suggests that opioids are strongly reinforcing to individuals with PTSD (69) and may initially treat the aversive symptoms. Improving social factors that decrease trauma, stress, and pain appear to be an important goal but are unlikely to be effective without reducing the overall supply and accessibility of opioids.

The biological theory of the opioid crisis may help inform future pharmacological interventions targeting key neurohormonal and/or microbial systems. An in-depth understanding of the neuroscience of addiction can also improve behavioral interventions targeting the cognitive aspects of relapse and recovery. Given our limited understanding of the biological underpinnings of OUD, one could assume that if society increased its levels of stress and depression, the epidemic could worsen due to more triggers for relapse (78). The biological theory of OUD opens possibilities for multiple interventions at the physiological level. Emerging data suggests that nutrition may be a useful adjunct for biological (169) as well as social (170) intervention. Given the links between impaired gastrointestinal health and neuroinflammation (145), targeted nutrition interventions may ameliorate neuroinflammation, which has been identified as a potential treatment for OUD (78). It has been argued that the microbiome is the link between person, public, and planetary health (184) and therefore we must consider environmental, psychosocial, and personal/nutritional factors implicated in gut dysbiosis. Much more research is needed on biological aspects of OUD that include nutrition-related factors which should consider the link between SES and access to food.

### Policy Interventions

While no one single policy intervention on opioid addiction has proven to be highly effective, several promising proposals have been made. Institutional level strategies (i.e., hospital) such as updated prescription guidelines for emergency rooms have decreased number of patients discharging with a prescription opioid by nearly 40% and sustained 2.5 years after the intervention (185). If the opioid crisis in the US is to be “solved” it will require a multilevel initiative engaging all sectors of the healthcare system (186). Prescription drug monitoring programs (PDMPs) (187) share data across states and represent a policy tool targeted toward providers, the broadest level of intervention in the socio-ecological model (188, 189). It has been shown that PDMP implementation is associated with reduced doctor shopping for prescription opiate painkillers, but PDMP utilization is not uniform across states, and has not yet been integrated into all EMRs (190, 191). There is good evidence that collaborative efforts with private health insurers can be successful in promoting best practices in opioid prescribing (192). This includes better training of pharmacists to detect and discuss drug misuse with patients (193). In 2016, Ohio passed a law that requires pharmacists to review a patient's PMDP history before dispensing a new controlled substance, encouraging denial to some patients and thereby taking a more proactive role in pain management (194). Additional policy level interventions and enforcement of current efforts will be critical.

### Treatment Implications

A trauma-focused treatment model typically involves empathy, curiosity, and trust (195). The current paradigm for OUD treatment is typically centered on psychotherapy in individual and group settings, in addition to psychiatry. Skills for distress tolerance and managing negative affect appear to be critical for maintaining sobriety. Other treatment approaches which consider neuroscience may lead to targeted treatments and better outcomes. Meanwhile, a purely medical approach to treatment (e.g., MAT alone) often fails to consider the importance of the patient-clinician relationship in the recovery process (195). Targeted treatments for individuals who are at heightened psychosocial and biological risk may benefit from the inclusion of enhanced treatment protocols such as gut-focused nutrition therapy. “Holistic” approaches including nutrition is not widely accepted but have been growing in popularity in the private sector. Leading experts are consistently calling for “research into new treatments for OUD” (196) but nutrition therapy has lagged behind. It has been argued that failure to address nutritional conditions can severely undermine treatment (197). In Los Angeles, nutrition services are offered at less than a third of SUD treatment centers (198). Our work has shown educational and culinary interventions can be effective despite operational challenges (199). Nutritional protocols for OUD have been described elsewhere (200) and specific group education topics for SUD treatment have also been recommended (201).

### Nutrition Interventions

Epidemiological research suggests that nutrient imbalance is a strong predictor of substance use and may be partially mediated by depression (202). Some authors have recommended dietary supplements for use in early recovery from heroin (203). It remains unknown how these supplements interact with MAT, and the reader is deferred to a more comprehensive review (200). Methadone maintenance patients have received nutritional counseling aimed at reducing diet-related morbidity but a lack of measurable changes (e.g., weight) reduce the scientific salience (204). Meanwhile, nutrition services provided within the Veterans Affairs health care system have been associated with significant improvements in treatment outcomes (205). A small study demonstrated improvements in self-reported abstinence following nutrition education during alcohol treatment (206). The concept of nutrition education has been successfully introduced into recovery programs within the prison system as means of improving overall wellness (207, 208). Improved eating patterns and reductions in waist circumference have occurred following educational and environmental interventions in male residential treatment settings (209, 210). Gender-specific approaches for women including education around body image reduced eating disorder symptomatology (211). Women in residential treatment have expressed increased concern about their food choices during recovery (212). Taken together, the potential for use of nutrition as part of a BPS framework for OUD treatment appears underutilized and poorly documented.

### Life Course Perspective

The Life Course Perspective (LCP) is not new to public health (213). The LCP puts a temporal element to the various factors in the BPS Perspective. LCP is a way of understanding human development and adaptation from an intergenerational approach considering all factors contributing to health outcomes, and how these factors accumulate over time. This concept has been described as a “biological embedding” of the environment and of one's lived experiences (214, 215). In the LCP, environment includes access to health and social services, which is directly linked to SES as well as cultural norms within that context. LCP includes complex concepts such as epigenetics and is therefore a much broader way of understanding health. Transgenerational inheritance of addiction-like behavior appears supported by epigenetic mechanisms (i.e., environmental exposure) over genetic factors (107). Furthermore, epigenetic modifications acquired in one generation can be inherited by the next generation and can involve behavioral or social transmission (107), including the transmission of trauma (216).

The LCP considers multiple pathways contributing to disease, at the biological level (genetic/epigenetic) but also emphasizing how social ties influence health behavior and how these accumulate throughout the life course (217). Meanwhile, LCP consider not only how disadvantage impacts health outcomes, but how cumulative advantage can play a role (218). In the case of OUD, social advantage may protect one from stress, or facilitate an individual receiving better treatment. LCP considers psychosocial mediators in the biological programing of health (219) and is therefore a major hub for recycling predictors of health outcomes in Figure 1.

### Biopsychosocial Perspective

The BPS Perspective incorporates all of the factors and levels discussed in this paper and recognizes environmental, psychosocial, biological, as well as their mediating factors. This single cohesive framework considers the interdependency of the entire system, drawing its conceptual roots from socio-ecological models (188, 189) including Ecosocial Theory (23). The BPS Perspective suggests the risk factors and protective factors that influence substance-seeking behavior at the individual and population level, and how they may impact health outcomes. Of particular importance in the opioid crisis appears to be the mediating role of life stressors, and possibly the role of gastrointestinal health. While genetics cannot be changed, epigenetics and the microbiome are both potential intervention targets by way of nutrition, albeit much slower than medication, and therefore difficult to measure (behavioral outcomes vs. biomarkers). Meanwhile, the reductionistic approach to generating high quality evidence contributes to the absence of evidence for more complex approaches, such as those discussed herein. Addressing the opioid crisis from all perspectives discussed herein should be considered a public health priority. More research is needed to determine if nutrition can be helpful.

### Future Directions

It will be important to identify aspects of an individual's neurochemistry which are modifiable by epigenetic and microbial mechanisms. With such strong evidence of overlapping pathways between drugs of abuse and food (220), it is surprising that food has not been investigated as a long-term modulator of reward pathways in humans. Meanwhile, authors from around the globe have suggested that nutrition interventions may be helpful in combating the opioid crisis (156, 198, 221). More evidence is needed before it will be recognized as a treatment modality. Specifically, it would be helpful to measure how nutrition interventions in early recovery can impact the gut microbiome, and how this can affect brain function (e.g., neuroinflammation) and thereby overall chances of recovery. With new measures and specific biomarkers of health status (e.g., allostatic load, microbiome, etc.), the BPS Perspective can be operationalized. This work may end up being conducted under the emerging field of “nutritional psychiatry” (222, 223). To date, gut-based nutrition interventions for OUD have not been investigated in humans but do appear to be timely.

## Conclusion

A multifactorial problem that requires effective collaboration across multiple disciplines at multiple levels has been described. The future of multidisciplinary BPS work will necessitate an understanding of health as a dynamic and integrated system. It has been emphasized that the potential for nutrition to be utilized as one facet of a BPS approach may improve recovery outcomes. At a minimum, we should consider nutritional screening at intake in OUD treatment programs. Going forward, we need policies that address access to opioids and pain management. The overdose epidemic should be viewed through the lens of community impact. Paramount are the social determinants of health, particularly given associations between social disadvantage and the lifetime accumulation of stress and trauma, as well as how social factors impact opioid use (224, 225) and nutritional status (226, 227). What happens in early life has profound consequences in adulthood, and what happens in one generation may hold significance for future generations. To combat the opioid epidemic, we cannot ignore either the social or the biological determinants of health. This paper adds to the voice of other authors that have called for a “biopsychosocial revolution” linking science and humanism (228). It is time to advocate for an integration of social and biological disciplines in order to better address the opioid tragedy. Collaborative efforts and partnerships across disciplines will be critical, and the field of public health nutrition appears ripe for leading the way.

## Data Availability

No datasets were generated or analyzed for this study.

## Author Contributions

The author confirms being the sole contributor of this work and has approved it for publication.

### Conflict of Interest Statement

The author declares that the research was conducted in the absence of any commercial or financial relationships that could be construed as a potential conflict of interest.

## References

[1] Rudd RA Aleshire N Zibbell JE Gladden MR. Increases in drug and opioid overdose deaths — United States, 2000–2014. MMWR Morb Mortal Wkly Rep. (2016) 64:1378–82. 10.15585/mmwr.mm6450a3 26720857

[2] Zoorob M. Polydrug epidemiology: benzodiazepine prescribing and the drug overdose epidemic in the United States. Pharmacoepidemiol Drug Saf. (2018) 27:541–9. 10.1002/pds.4417 29537112

[3] Hsu DJ McCarthy EP Stevens JP Mukamal KJ. Hospitalizations, costs and outcomes associated with heroin and prescription opioid overdoses in the United States 2001–12. Addiction. (2017) 112:1558–64. 10.1111/add.13795 28191702 PMC5544564

[4] Maxwell J The pain reliever and heroin epidemic in the United States: shifting winds in the perfect storm. J Addict Dis. (2015) 34:127–40. 10.1080/10550887.2015.1059667 26106929

[5] Lee JD Friedmann PD Kinlock TW Nunes EV Boney TY Hoskinson RA. Extended-release naltrexone to prevent opioid relapse in criminal justice offenders. N Engl J Med. (2016) 374:1232–42. 10.1056/NEJMoa1505409 27028913 PMC5454800

[6] Nunes EV Gordon M Friedmann PD Fishman MJ Lee JD Chen DT. Relapse to opioid use disorder after inpatient treatment: protective effect of injection naltrexone. J Subst Abuse Treat. (2018) 85:49–55. 10.1016/j.jsat.2017.04.016 28473233 PMC5755382

[7] Kadam M Sinha A Nimkar S Matcheswalla Y Sousa A. A comparative study of factors associated with relapse in alcohol dependence and opioid dependence. Indian J Psychol Med. (2017) 39:627. 10.4103/IJPSYM.IJPSYM_356_17 29200559 PMC5688890

[8] Wegman MP Altice FL Kaur S Rajandaran V Osornprasop S Wilson D. Relapse to opioid use in opioid-dependent individuals released from compulsory drug detention centres compared with those from voluntary methadone treatment centres in Malaysia: a two-arm, prospective observational study. Lancet Glob Health. (2017) 5:e198–207. 10.1016/S2214-109X(16)30303-5 27964869 PMC5657487

[9] Rudd RA Seth P David F Scholl L. Increases in drug and opioid-involved overdose deaths — United States, 2010–2015. MMWR Morb Mortal Wkly Rep. (2016) 65:1445–52. 10.15585/mmwr.mm655051e1 28033313

[10] Egervari G Ciccocioppo R Jentsch DJ Hurd YL. Shaping vulnerability to addiction – the contribution of behavior, neural circuits and molecular mechanisms. Neurosci Biobehav Rev. (2018) 85:117–25. 10.1016/j.neubiorev.2017.05.019 28571877 PMC5708151

[11] Bart G. Maintenance medication for opiate addiction: the foundation of recovery. J Addict Dis. (2012) 31:207–25. 10.1080/10550887.2012.694598 22873183 PMC3411273

[12] Sokol R LaVertu AE Morrill D Albanese C Schuman-Olivier Z. Group-based treatment of opioid use disorder with buprenorphine: a systematic review. J Subst Abuse Treat. (2018) 84:78–87. 10.1016/j.jsat.2017.11.003 29195596

[13] Pergolizzi JV LeQuang J Berger GK Raffa RB. The Basic Pharmacology of opioids informs the opioid discourse about misuse and abuse: a review. Pain Ther. (2017) 6:1–16. 10.1007/s40122-017-0068-3 28341939 PMC5447545

[14] Lyapustina T Castillo R Omaki E Shields W McDonald E Rothman R The contribution of the emergency department to opioid pain reliever misuse and diversion: a critical review. Pain Pract. (2017) 17:1097–104. 10.1111/papr.12568 28226416

[15] Weiner SG Raja AS Bittner JC Curtis KM Weimersheimer P Hasegawa K. Opioid-related policies in new england emergency departments. Acad Emerg Med. (2016) 23:1086–90. 10.1111/acem.12992 27098615

[16] Martin BC Fan M-Y Edlund MJ DeVries A Braden J Sullivan MD. Long-term chronic opioid therapy discontinuation rates from the TROUP study. J Gen Intern Med. (2011) 26:1450–7. 10.1007/s11606-011-1771-0 21751058 PMC3235603

[17] Martins SS Segura LE Santaella-Tenorio J Perlmutter A Fenton MC Cerdá M. Prescription opioid use disorder and heroin use among 12-34 year-olds in the United States from 2002 to 2014. Addict Behav. (2017) 65:236–41. 10.1016/j.addbeh.2016.08.033 27614657 PMC5140701

[18] Cicero TJ Ellis MS. Abuse-deterrent formulations and the prescription opioid abuse epidemic in the United States: lessons learned from oxycontin. JAMA Psychiatry. (2015) 72:424–30. 10.1001/jamapsychiatry.2014.3043 25760692

[19] Schwartz RP Gryczynski J O'Grady KE Arfstein J Warren G Olsen Y. Opioid agonist treatments and heroin overdose deaths in Baltimore, Maryland, 1995–2009. Am J Public Health. (2013) 103:917–22. 10.2105/AJPH.2012.301049 23488511 PMC3670653

[20] Engel G. The clinical application of the biopsychosocial model. Am J Psychiatry. (1980) 137:535–44. 10.1176/ajp.137.5.535 7369396

[21] Rousseau D General systems theory: its present and potential. Syst Res Behav Sci. (2015) 32:522–33. 10.1002/sres.2354

[22] Fava G Sonino N. Psychosomatic medicine: emerging trends and perspectives. Psychother Psychosom. (2000) 69:184–97. 10.1159/000012393 10867586

[23] Krieger N. Epidemiology and the web of causation: has anyone seen the spider? Soc Sci Med. (1994) 39:887–903. 10.1016/0277-9536(94)90202-X 7992123

[24] Waite LJ Levinson W Lindau S Laumann EO. Synthesis of scientific disciplines in pursuit of health: the interactive biopsychosocial model. Perspect Biol Med. (2003) 46:S74–86. 10.1353/pbm.2003.0069 14563076 PMC1201376

[25] Roux AV Integrating social and biologic factors in health research: a systems view. Ann Epidemiol. (2007) 17:569–74. 10.1016/j.annepidem.2007.03.001 17553703

[26] Frances A. Resuscitating the biopsychosocial model. Lancet Psychiatry. (2014) 1:496–7. 10.1016/S2215-0366(14)00058-3 26361297

[27] Griffiths M A ‘components’ model of addiction within a biopsychosocial framework. J Subst Use. (2009) 10:191–7. 10.1080/14659890500114359

[28] Blendon RJ Benson JM. The public and the opioid-abuse epidemic. New Engl J Med. (2018) 378:407–11. 10.1056/NEJMp1714529 29298128

[29] Dowell D Haegerich TM Chou R. CDC guideline for prescribing opioids for chronic pain—United States, 2016. JAMA. (2016) 315:1624. 10.1001/jama.2016.1464 26977696 PMC6390846

[30] Hays GP Mycyk MB. Confronting the opioid crisis by taking a long look in the mirror … and at our peers. Acad Emerg Med. (2018) 25:594–6. 10.1111/acem.13397 29498140

[31] Cicero TJ Ellis MS Kasper ZA. Increased use of heroin as an initiating opioid of abuse. Addict Behav. (2017) 74:63–6. 10.1016/j.addbeh.2017.05.030 28582659

[32] Jones CM. Heroin use and heroin use risk behaviors among nonmedical users of prescription opioid pain relievers- United States, 2002-2004 and 2008-2010. Drug Alcohol Depend. (2013) 132:95–100. 10.1016/j.drugalcdep.2013.01.007 23410617

[33] Kertesz SG. Turning the tide or riptide? The changing opioid epidemic. Subst Abus. (2016) 38:3–8. 10.1080/08897077.2016.1261070 27858590

[34] Hser Y-I Mooney LJ Saxon AJ Miotto K Bell DS Huang D. Chronic pain among patients with opioid use disorder: results from electronic health records data. J Subst Abuse Treat. (2017) 77:26–30. 10.1016/j.jsat.2017.03.006 28476267 PMC5424616

[35] Haegerich TM Paulozzi LJ Manns BJ Jones CM. What we know, and don't know, about the impact of state policy and systems-level interventions on prescription drug overdose. Drug Alcohol Depend. (2014) 145:34–47. 10.1016/j.drugalcdep.2014.10.001 25454406 PMC6557270

[36] Rickles NM Huang AL Gunther MB Chan WJ. An opioid dispensing and misuse prevention algorithm for community pharmacy practice. Res Soc Adm Pharm. (2018). 10.1016/j.sapharm.2018.02.004. [Epub ahead of print]. 29525483

[37] Arora NS Marcotte KM Hopper JA. Reducing opioid misuse among adolescents through physician education. Subst Abus. (2018) 39:6–8. 10.1080/08897077.2017.1356788 28723248

[38] Piper BJ Desrosiers CE Fisher HC McCall KL Nichols SD. A new tool to tackle the opioid epidemic: description, utility, and results from the maine diversion alert program. Pharmacotherapy. (2017) 37:791–8. 10.1002/phar.1952 28543168 PMC5693423

[39] Marinova Z Maercker A. Biological correlates of complex posttraumatic stress disorder—state of research and future directions. Eur J Psychotraumatol. (2015) 6:25913. 10.3402/ejpt.v6.25913 25887894 PMC4401823

[40] Epstein LH Salvy SJ Carr KA Dearing KK Bickel WK. Food reinforcement, delay discounting and obesity. Physiol Behav. (2010) 100:438–45. 10.1016/j.physbeh.2010.04.029 20435052 PMC9632539

[41] Joynt M Train MK Robbins BW Halterman JS Caiola E Fortuna RJ. The impact of neighborhood socioeconomic status and race on the prescribing of opioids in emergency departments throughout the United States. J Gen Intern Med. (2013) 28:1604–10. 10.1007/s11606-013-2516-z 23797920 PMC3832731

[42] Koob GF Schulkin J. Addiction and stress: an allostatic view. Neurosci Biobehav Rev. (2018). 10.1016/j.neubiorev.2018.09.008. [Epub ahead of print]. 30227143

[43] Amundsen EJ. Drug-related causes of death: socioeconomic and demographic characteristics of the deceased. Scand J Public Health. (2015) 43:571–9. 10.1177/1403494815585909 25969166

[44] Bossaerts P Murawski C From behavioural economics to neuroeconomics to decision neuroscience: the ascent of biology in research on human decision making. Curr Opin Behav Sci. (2015) 5:37–42. 10.1016/j.cobeha.2015.07.001

[45] Bickel WK Moody L Higgins ST. Some current dimensions of the behavioral economics of health-related behavior change. Prevent Med. (2016) 92:16–23. 10.1016/j.ypmed.2016.06.002 27283095 PMC5085840

[46] Bickel WK Quisenberry AJ Moody L Wilson GA. Therapeutic opportunities for self-control repair in addiction and related disorders. Clin Psychol Sci. (2014) 3:140–53. 10.1177/2167702614541260 25664226 PMC4314724

[47] Bickel WK Marsch LA. Toward a behavioral economic understanding of drug dependence: delay discounting processes. Addiction. (2001) 96:73–86. 10.1046/j.1360-0443.2001.961736.x 11177521

[48] Bickel WK DeGrandpre RJ Higgins ST. Behavioral economics: a novel experimental approach to the study of drug dependence. Drug Alcohol Depend. (1993) 33:173–92. 10.1016/0376-8716(93)90059-Y 8261882

[49] MacKillop J. The behavioral economics and neuroeconomics of alcohol use disorders. Alcohol Clin Exp Res. (2016) 40:672–85. 10.1111/acer.13004 26993151 PMC4846981

[50] Stojek M MacKillop J. Relative reinforcing value of food and delayed reward discounting in obesity and disordered eating: a systematic review. Clin Psychol Rev. (2017) 55:1–11. 10.1016/j.cpr.2017.04.007 28478269

[51] Rubin LP. Maternal and pediatric health and disease: integrating biopsychosocial models and epigenetics. Pediatr Res. (2015) 79:127–35. 10.1038/pr.2015.203 26484619

[52] Gebauer S Salas J Scherrer JF. Neighborhood socioeconomic status and receipt of opioid medication for new back pain diagnosis. J Am Board Fam Med. (2017) 30:775–83. 10.3122/jabfm.2017.06.170061 29180552

[53] Jones CM Logan J Gladden R Bohm MK. Vital signs: demographic and substance use trends among heroin users - United States, 2002-2013. MMWR Morb Mortal Wkly Rep. (2015) 64:719–25. 26158353 PMC4584844

[54] Marshall JR Gassner SF Anderson CL Cooper RJ Lotfipour S Chakravarthy B. Socioeconomic and geographical disparities in prescription and illicit opioid-related overdose deaths in Orange County, California, from 2010–2014. Subst Abuse. (2018) 40:80–6. 10.1080/08897077.2018.1442899 29465301

[55] Campbell CI Bahorik AL Velduisen P Weisner C Rubinstein AL Ray TG. Use of a prescription opioid registry to examine opioid misuse and overdose in an integrated health system. Prev Med. (2018) 110:31–7. 10.1016/j.ypmed.2018.01.019 29410132 PMC6034705

[56] Jones CM. Trends and key correlates of prescription opioid injection misuse in the United States. Addict Behav. (2018) 78:145–52. 10.1016/j.addbeh.2017.10.018 29175290

[57] Guy GP Zhang K Bohm MK Losby J Lewis B Young R. Vital signs: changes in opioid prescribing in the United States, 2006-2015. MMWR Morbid Mortal Wkly Rep. (2017) 66:697–704. 10.15585/mmwr.mm6626a4 28683056 PMC5726238

[58] Hand DJ Short VL Abatemarco DJ. Substance use, treatment, and demographic characteristics of pregnant women entering treatment for opioid use disorder differ by United States census region. J Subst Abuse Treat. (2017) 76:58–63. 10.1016/j.jsat.2017.01.011 28161143

[59] Bickel WK Johnson MW Koffarnus MN MacKillop J Murphy JG. The behavioral economics of substance use disorders: reinforcement pathologies and their repair. Annu Rev Clin Psychol. (2014) 10:641–77. 10.1146/annurev-clinpsy-032813-153724 24679180 PMC4501268

[60] Ressler KJ. Molecular signatures of stress and posttraumatic stress disorder: an overview. Biol Psychiatry. (2018) 83:792–4. 10.1016/j.biopsych.2018.03.007 29685184

[61] Langford DJ Theodore BR Balsiger D Tran C Doorenbos AZ Tauben DJ Number and type of Post-Traumatic Stress Disorder (PTSD) symptom domains are associated with patient-reported outcomes in patients with chronic pain. J Pain. (2018) 19:506–14. 10.1016/j.jpain.2017.12.262 29307748 PMC5927843

[62] Colvonen PJ Ellison J Haller M Norman SB. Examining insomnia and PTSD over time in veterans in residential treatment for substance use disorders and PTSD. Behav Sleep Med. (2018) 17:524–35. 10.1080/15402002.2018.1425869 29364693 PMC6645391

[63] Seal KH Maguen S Bertenthal D Batki SL Striebel J Stein MB. Observational evidence for buprenorphine's impact on posttraumatic stress symptoms in veterans with chronic pain and opioid use disorder. J Clin Psychiatry. (2016) 77:1182–8. 10.4088/JCP.15m09893 27035058

[64] Stark EA Parsons CE Hartevelt TJ Charquero-Ballester M McManners H Ehlers A. Post-traumatic stress influences the brain even in the absence of symptoms: a systematic, quantitative meta-analysis of neuroimaging studies. Neurosci Biobehav Rev. (2015) 56:207–21. 10.1016/j.neubiorev.2015.07.007 26192104

[65] Hassan AN Foll B Imtiaz S Rehm J. The effect of post-traumatic stress disorder on the risk of developing prescription opioid use disorder: results from the National Epidemiologic Survey on Alcohol and Related Conditions III. Drug Alcohol Depend. (2017) 179:260–6. 10.1016/j.drugalcdep.2017.07.012 28818717

[66] Smith KZ Smith PH Cercone SA McKee SA Homish GG. Past year non-medical opioid use and abuse and PTSD diagnosis: interactions with sex and associations with symptom clusters. Addict Behav. (2016) 58:167–74. 10.1016/j.addbeh.2016.02.019 26946448 PMC4808454

[67] Patel RS Elmaadawi A Nasr S Haskin J. Comorbid post-traumatic stress disorder and opioid dependence. Cureus. (2017) 9:e1647. 10.7759/cureus.1647 29142795 PMC5669522

[68] Nawijn L van Zuiden M Frijling JL Koch S Veltman DJ Olff M. Reward functioning in PTSD: a systematic review exploring the mechanisms underlying anhedonia. Neurosci Biobehav Rev. (2015) 51:189–204. 10.1016/j.neubiorev.2015.01.019 25639225

[69] Danovitch I. Post-traumatic stress disorder and opioid use disorder: a narrative review of conceptual models. J Addict Dis. (2016) 35:1–11. 10.1080/10550887.2016.1168212 27010975

[70] Anda RF Whitfield CL Felitti VJ Chapman D Edwards VJ Dube SR. Adverse childhood experiences, alcoholic parents, and later risk of alcoholism and depression. Psychiatr Serv. (2002) 53:1001–9. 10.1176/appi.ps.53.8.1001 12161676

[71] Dube SR Felitti VJ Dong M Chapman DP Giles WH Anda RF. Childhood abuse, neglect, and household dysfunction and the risk of illicit drug use: the adverse childhood experiences study. Pediatrics. (2003) 111:564–72. 10.1542/peds.111.3.564 12612237

[72] Felitti VJ Anda RF Nordenberg D Williamson DF Spitz AM Edwards V. Relationship of childhood abuse and household dysfunction to many of the leading causes of death in adults the Adverse Childhood Experiences (ACE) study. Am J Prevent Med. (1998) 14:245–58. 10.1016/S0749-3797(98)00017-8 9635069

[73] Stein MD Conti MT Kenney S Anderson BJ Flori JN Risi MM. Adverse childhood experience effects on opioid use initiation, injection drug use, and overdose among persons with opioid use disorder. Drug Alcohol Depend. (2017) 179:325–9. 10.1016/j.drugalcdep.2017.07.007 28841495 PMC5599365

[74] Austin AE Shanahan ME Association of childhood abuse and neglect with prescription opioid misuse: examination of mediation by adolescent depressive symptoms and pain. Child Youth Serv Rev. (2018) 86:84–93. 10.1016/j.childyouth.2018.01.023

[75] Austin AE Shanahan ME Zvara BJ. Association of childhood abuse and prescription opioid use in early adulthood. Addict Behav. (2018) 76:265–9. 10.1016/j.addbeh.2017.08.033 28869906

[76] Mersky JP Topitzes J Reynolds AJ. Impacts of adverse childhood experiences on health, mental health, and substance use in early adulthood: a cohort study of an urban, minority sample in the U.S. Child Abuse Neglect. (2013) 37:917–25. 10.1016/j.chiabu.2013.07.011 23978575 PMC4090696

[77] Cook A Spinazzola J Ford J Lanktree C Blaustein M Cloitre M Complex trauma in children and adolescents. Psychiatr Ann. (2005) 35:390–8. 10.3928/00485713-20050501-05

[78] Evans C Cahill C. Neurobiology of opioid dependence in creating addiction vulnerability. F1000Research. (2016) 5:F1000 Faculty Rev-1748. 10.12688/f1000research.8369.1 27508068 PMC4955026

[79] Eitan S Emery MA Bates MLS Horrax C. Opioid addiction: who are your real friends? Neurosci Biobehav Rev. (2017) 83:697–712. 10.1016/j.neubiorev.2017.05.017 28552458

[80] Ford JA Sacra S Yohros A. Neighborhood characteristics and prescription drug misuse among adolescents: the importance of social disorganization and social capital. Int J Drug Policy. (2017) 46:47–53. 10.1016/j.drugpo.2017.05.001 28609748

[81] Taylor SE Repetti RL Seeman T. Health psychology: what is an unhealthy environment and how does it get under the skin? Psychology. (1997) 48:411–47. 10.1146/annurev.psych.48.1.411 9046565

[82] Khantzian EJ. The Self-Medication Hypothesis of Addictive Disorders: Focus on Heroin and Cocaine Dependence. New York, NY: Springer (1987). 10.1176/ajp.142.11.1259 3904487

[83] McCrory EJ Viding E. The theory of latent vulnerability: reconceptualizing the link between childhood maltreatment and psychiatric disorder. Dev Psychopathol. (2015) 27:493–505. 10.1017/S0954579415000115 25997767

[84] Puetz VB McCrory E. Exploring the relationship between childhood maltreatment and addiction: a review of the neurocognitive evidence. Curr Addict Rep. (2015) 2:318–25. 10.1007/s40429-015-0073-8 26550550 PMC4628081

[85] Black AC Cooney NL Sartor CE Arias AJ Rosen MI. Impulsivity interacts with momentary PTSD symptom worsening to predict alcohol use in male veterans. Am J Drug Alcohol Abuse. (2018) 44:524–31. 10.1080/00952990.2018.1454935 29641264 PMC6172135

[86] Weiss NH Tull MT Lavender J Gratz KL. Role of emotion dysregulation in the relationship between childhood abuse and probable PTSD in a sample of substance abusers. Child Abuse Neglect. (2013) 37:944–54. 10.1016/j.chiabu.2013.03.014 23643388

[87] Mirhashem R Allen HC Adams ZW van Stolk-Cooke K Legrand A Price M. The intervening role of urgency on the association between childhood maltreatment, PTSD, and substance-related problems. Addict Behav. (2017) 69:98–103. 10.1016/j.addbeh.2017.02.012 28219827 PMC5384831

[88] Volkow ND Koob G. Brain disease model of addiction: why is it so controversial? Lancet Psychiatry. (2015) 2:677–9. 10.1016/S2215-0366(15)00236-9 26249284 PMC4556943

[89] Longo DL Volkow ND Koob GF McLellan TA Neurobiologic advances from the brain disease model of addiction. New Engl J Med. (2016) 374:363–71. 10.1056/NEJMra1511480 26816013 PMC6135257

[90] Volkow ND Boyle M. Neuroscience of addiction: relevance to prevention and treatment. Am J Psychiatry. (2018) 175:729–40. 10.1176/appi.ajp.2018.17101174 29690790

[91] Moeller SJ London ED Northoff G. Neuroimaging markers of glutamatergic and GABAergic systems in drug addiction: relationships to resting-state functional connectivity. Neurosci Biobehav Rev. (2016) 61:35–52. 10.1016/j.neubiorev.2015.11.010 26657968 PMC4731270

[92] Moeller SJ Paulus MP. Toward biomarkers of the addicted human brain: using neuroimaging to predict relapse and sustained abstinence in substance use disorder. Prog Neuropsychopharmacol Biol Psychiatry. (2018) 80(Pt B):143–54. 10.1016/j.pnpbp.2017.03.003 28322982 PMC5603350

[93] Jasinska AJ Stein EA Kaiser J Naumer MJ Yalachkov Y. Factors modulating neural reactivity to drug cues in addiction: a survey of human neuroimaging studies. Neurosci Biobehav Rev. (2014) 38:1–16. 10.1016/j.neubiorev.2013.10.013 24211373 PMC3913480

[94] Jentsch DJ Ashenhurst JR Cervantes CM Groman SM James AS Pennington ZT. Dissecting impulsivity and its relationships to drug addictions. Ann NY Acad Sci. (2017) 1327:1–26. 10.1111/nyas.12388 24654857 PMC4360991

[95] Finan PH Remeniuk B Dunn KE. The risk for problematic opioid use in chronic pain: what can we learn from studies of pain and reward? Prog Neuropsychopharmacol Biol Psychiatry. (2017) 87(Pt B):255–62. 10.1016/j.pnpbp.2017.07.029 28778406 PMC5821601

[96] Felger JC Treadway MT. Inflammation effects on motivation and motor activity: role of dopamine. Neuropsychopharmacology. (2016) 42:216–41. 10.1038/npp.2016.143 27480574 PMC5143486

[97] Wassum KM Greenfield VY Linker KE Maidment NT Ostlund SB. Inflated reward value in early opiate withdrawal. Addict Biol. (2016) 21:221–33. 10.1111/adb.12172 25081350 PMC4312551

[98] Lubman DI Garfield J Gwini SM Cheetham A Cotton SM Yücel M. Dynamic associations between opioid use and anhedonia: a longitudinal study in opioid dependence. J Psychopharmacol. (2018) 32:957–64. 10.1177/0269881118791741 30130143

[99] Schulte E Gearhardt AN. Associations of food addiction in a sample recruited to be nationally representative of the United States. Eur Eat Disord Rev. (2018) 26:112–9. 10.1002/erv.2575 29266583

[100] Schulte E Sonneville KR Gearhardt AN. Subjective experiences of highly processed food consumption in individuals with food addiction. Psychol Addict Behav. (2019) 33:144–53. 10.1037/adb0000441 30628798

[101] Gorwood P Strat Y Ramoz N Dubertret C Moalic J-M Simonneau M. Genetics of dopamine receptors and drug addiction. Hum Genet. (2012) 131:803–22. 10.1007/s00439-012-1145-7 22350797

[102] Gray JC MacKillop J Weafer J Hernandez KM Gao J Palmer AA. Genetic analysis of impulsive personality traits: examination of a priori candidates and genome-wide variation. Psychiatry Res. (2018) 259:398–404. 10.1016/j.psychres.2017.10.047 29120849 PMC5742029

[103] Blum K Chen AL Oscar-Berman M Chen TJ Lubar J White N. Generational association studies of dopaminergic genes in Reward Deficiency Syndrome (RDS) Subjects: selecting appropriate phenotypes for reward dependence behaviors. Int J Environ Res Public Health. (2011) 8:4425–59. 10.3390/ijerph8124425 22408582 PMC3290972

[104] Blum K Sheridan P Wood R Braverman E Chen T Cull JG. The D2 dopamine receptor gene as a determinant of reward deficiency syndrome. J R Soc Med. (1996) 89:396–400. 10.1177/014107689608900711 8774539 PMC1295855

[105] Comings DE Muhleman D Gysin R. Dopamine D2 receptor (DRD2) gene and susceptibility to posttraumatic stress disorder: a study and replication. Biol Psychiatry. (1996) 40:368–72. 10.1016/0006-3223(95)00519-6 8874837

[106] Blum K Oscar-Berman M Demetrovics Z Barh D Gold MS. Genetic Addiction Risk Score (GARS): molecular neurogenetic evidence for predisposition to Reward Deficiency Syndrome (RDS). Mol Neurobiol. (2014) 50:765–96. 10.1007/s12035-014-8726-5 24878765 PMC4225054

[107] Vassoler FM Sadri-Vakili G. Mechanisms of transgenerational inheritance of addictive-like behaviors. Neuroscience. (2014) 264:198–206. 10.1016/j.neuroscience.2013.07.064 23920159 PMC3872494

[108] Rustichini A. Neuroeconomics: what have we found, and what should we search for. Curr Opin Neurobiol. (2009) 19:672–7. 10.1016/j.conb.2009.09.012 19896360

[109] O'Doherty JP Camerer CC Editorial overview: neuroeconomics. Curr Opin Behav Sci. (2015) 5:v–viii. 10.1016/j.cobeha.2015.10.004

[110] Monterosso J Piray P Luo S. Neuroeconomics and the study of addiction. Biol Psychiatry. (2012) 72:107–12. 10.1016/j.biopsych.2012.03.012 22520343

[111] Sweis B Thomas MJ Redish DA. Beyond simple tests of value: measuring addiction as a heterogeneous disease of computation-specific valuation processes. Learn Memory. (2018) 25:501–12. 10.1101/lm.047795.118 30115772 PMC6097760

[112] Bickel WK Moody L Quisenberry AJ Ramey CT Sheffer CE. A Competing Neurobehavioral Decision Systems model of SES-related health and behavioral disparities. Prevent Med. (2014) 68:37–43. 10.1016/j.ypmed.2014.06.032 25008219 PMC4253853

[113] Camerer CF. Neuroeconomics: opening the gray box. Neuron. (2008) 60:416–9. 10.1016/j.neuron.2008.10.027 18995815

[114] Wei L-N. Epigenetic control of the expression of opioid receptor genes. Epigenetics. (2008) 3:119–21. 10.4161/epi.3.3.6296 18497574

[115] Vassoler FM Wright SJ Byrnes EM. Exposure to opiates in female adolescents alters mu opiate receptor expression and increases the rewarding effects of morphine in future offspring. Neuropharmacology. (2016) 103:112–21. 10.1016/j.neuropharm.2015.11.026 26700246 PMC4755844

[116] Denhardt DT. Effect of stress on human biology: epigenetics, adaptation, inheritance, and social significance. J Cell Physiol. (2018) 233:1975–84. 10.1002/jcp.25837 28158904

[117] Cao-Lei L Rooij SR King S Matthews SG Metz GAS Roseboom TJ. Prenatal stress and epigenetics. Neurosci Biobehav Rev. (2017). 10.1016/j.neubiorev.2017.05.016. [Epub ahead of print]. 28528960

[118] Chan JC Nugent BM Bale TL. Parental advisory: maternal and paternal stress can impact offspring neurodevelopment. Biol Psychiatry. (2018) 83:886–894. 10.1016/j.biopsych.2017.10.005 29198470 PMC5899063

[119] Van den Bergh B van den Heuvel MI Lahti M Braeken M de Rooij SR Entringer S. Prenatal developmental origins of behavior and mental health: the influence of maternal stress in pregnancy. Neurosci Biobehav Rev. (2017). 10.1016/j.neubiorev.2017.07.003. [Epub ahead of print]. 28757456

[120] Steiger H Thaler L. Eating disorders, gene-environment interactions and the epigenome: roles of stress exposures and nutritional status. Physiol Behav. (2016) 162:181–5. 10.1016/j.physbeh.2016.01.041 26836275

[121] Montalvo-Martínez L Maldonado-Ruiz R Cárdenas-Tueme M Reséndez-Pérez D Camacho A. Maternal overnutrition programs central inflammation and addiction-like behavior in offspring. Biomed Res Int. (2018) 2018:8061389. 10.1155/2018/8061389 30027100 PMC6031166

[122] Wiss DA Criscitelli K Gold M Avena N. Preclinical evidence for the addiction potential of highly palatable foods: current developments related to maternal influence. Appetite. (2017) 115:19–27. 10.1016/j.appet.2016.12.019 27989563

[123] Camacho A Montalvo-Martinez L Cardenas-Perez RE Fuentes-Mera L Garza-Ocañas L. Obesogenic diet intake during pregnancy programs aberrant synaptic plasticity and addiction-like behavior to a palatable food in offspring. Behav Brain Res. (2017) 330:46–55. 10.1016/j.bbr.2017.05.014 28487223

[124] van de Guchte M Blottière HM Doré J. Humans as holobionts: implications for prevention and therapy. Microbiome. (2018) 6:81. 10.1186/s40168-018-0466-8 29716650 PMC5928587

[125] Verdino J. The third tier in treatment: attending to the growing connection between gut health and emotional well-being. Health Psychol Open. (2017) 4:1–5. 10.1177/2055102917724335 29379615 PMC5779924

[126] Zalar B Haslberger A Peterlin B. The role of microbiota in depression - a brief review. Psychiat Danub. (2018) 30:136–41. 10.24869/spsih.2018.136 29930222

[127] Wallace CJ Milev R The effects of probiotics on depressive symptoms in humans: a systematic review. Ann Gen Psychiatry. (2017) 16:14 10.1186/s12991-017-0138-2 28239408 PMC5319175

[128] Huang R Wang K Hu J. Effect of probiotics on depression: a systematic review and meta-analysis of randomized controlled trials. Nutrients. (2016) 8:483. 10.3390/nu8080483 27509521 PMC4997396

[129] Taylor AM Holscher HD. A review of dietary and microbial connections to depression, anxiety, and stress. Nutr Neurosci. (2018) 1–14. 10.1080/1028415X.2018.1493808. [Epub ahead of print]. 29985786

[130] Leclercq S Forsythe P Bienenstock J. Posttraumatic stress disorder: does the gut microbiome hold the key? Can J Psychiatry. (2016) 61:204–13. 10.1177/0706743716635535 27254412 PMC4794957

[131] Cryan JF. Stress and the microbiota-gut-brain axis. Can J Psychiatry. (2016) 61:201–3. 10.1177/0706743716635538 27254411 PMC4794959

[132] Herd P Palloni A Rey F Dowd JB Social and population health science approaches to understand the human microbiome. Nat Hum Behav. (2018) 2:808–15. 10.1038/s41562-018-0452-y PMC6711373 31457107

[133] Shechter A Schwartz GJ. Gut–brain nutrient sensing in food reward. Appetite. (2018) 122:32–5. 10.1016/j.appet.2016.12.009 28007490 PMC5776705

[134] van de Wouw M Schellekens H Dinan TG Cryan JF. Microbiota-gut-brain axis: modulator of host metabolism and appetite. J Nutr. (2017) 147:727–45. 10.3945/jn.116.240481 28356427

[135] Vanamala J Knight R Spector TD. Can your microbiome tell you what to eat? Cell Metab. (2015) 22:960–1. 10.1016/j.cmet.2015.11.009 26636494

[136] Lerner A Neidhöfer S Matthias T. The gut microbiome feelings of the brain: a perspective for non-microbiologists. Microorganisms. (2017) 5:66. 10.3390/microorganisms5040066 29023380 PMC5748575

[137] Johnson K Foster KR. Why does the microbiome affect behaviour? Nat Rev Microbiol. (2018). 16:1. 10.1038/s41579-018-0014-3 29691482

[138] Lam YY Maguire S Palacios T Caterson ID. Are the gut bacteria telling us to eat or not to eat? Reviewing the role of gut microbiota in the etiology, disease progression and treatment of eating disorders. Nutrients. (2017) 9:602. 10.3390/nu9060602 28613252 PMC5490581

[139] Cussotto S Sandhu KV Dinan TG Cryan JF. The neuroendocrinology of the microbiota-gut-brain axis: a behavioural perspective. Front Neuroendocrinol. (2018) 51:002. 10.1016/j.yfrne.2018.04.002 29753796

[140] Tripathi A Debelius J Brenner DA Karin M Loomba R Schnabl B The gut–liver axis and the intersection with the microbiome. Nat Rev Gastroenterol Hepatol. (2018) 15:1 10.1038/s41575-018-0011-z 29748586 PMC6319369

[141] Bishehsari F Magno E Swanson G Desai V Voigt R Forsyth C. Alcohol and gut-derived inflammation. Alcohol Res Curr Rev. (2017) 38:163–71. 28988571 10.35946/arcr.v38.2.02 PMC5513683

[142] de Timary P Stärkel P lzenne N Leclercq S. A role for the peripheral immune system in the development of alcohol use disorders? Neuropharmacology. (2017) 122:148–60. 10.1016/j.neuropharm.2017.04.013 28400259

[143] Tsuruya A Kuwahara A Saito Y Yamaguchi H Tsubo T Suga S. Ecophysiological consequences of alcoholism on human gut microbiota: implications for ethanol-related pathogenesis of colon cancer. Sci Rep. (2016) 6:srep27923. 10.1038/srep27923 27295340 PMC4904738

[144] Leclercq S Matamoros S Cani PD Neyrinck AM Jamar F Stärkel P. Intestinal permeability, gut-bacterial dysbiosis, and behavioral markers of alcohol-dependence severity. Proc Natl Acad Sci USA. (2014) 111:E4485–93. 10.1073/pnas.1415174111 25288760 PMC4210345

[145] Leclercq S Cani PD Neyrinck AM Stärkel P Jamar F Mikolajczak M. Role of intestinal permeability and inflammation in the biological and behavioral control of alcohol-dependent subjects. Brain Behav Immun. (2012) 26:911–8. 10.1016/j.bbi.2012.04.001 22521198

[146] Loveland D Driscoll H. Examining attrition rates at one specialty addiction treatment provider in the United States: a case study using a retrospective chart review. Subst Abus Treat Prev Policy. (2014) 9:41. 10.1186/1747-597X-9-41 25255797 PMC4189207

[147] Zador D Wall LP Webster I. High sugar intake in a group of women on methadone maintenance in South Western Sydney, Australia. Addiction. (1996) 91:1053–61. 10.1111/j.1360-0443.1996.tb03601.x 8688819

[148] Nolan L Scagnelli L. Preference for sweet foods and higher body mass index in patients being treated in long-term methadone maintenance. Subst Use Misuse. (2007) 42:1555–66. 10.1080/10826080701517727 17918026

[149] Morabia A Fabre J Chee E Zeger S Orsat E Robert A. Diet and opiate addiction: A quantitative assessment of the diet of non-institutionalized opiate addicts. Br J Addict. (1989) 84:173–80. 10.1111/j.1360-0443.1989.tb00566.x 2720181

[150] Alves D Costa A Custodio D Natario L Ferro-Lebres V Andrade F Housing and employment situation, body mass index and dietary habits of heroin addicts in methadone maintenance treatment. Heroin Addict Relat Clin Probl. (2011) 13:11–4.

[151] Neale J Nettleton S Pickering L Fischer J. Eating patterns among heroin users: a qualitative study with implications for nutritional interventions. Addiction. (2012) 107:635–41. 10.1111/j.1360-0443.2011.03660.x 21933297

[152] Canan F Karaca S Sogucak S Gecici O Kuloglu M. Eating disorders and food addiction in men with heroin use disorder: a controlled study. Eat Weight Disord. (2017) 22:249–57. 10.1007/s40519-017-0378-9 28434177

[153] Cowan J Devine C. Food, eating, and weight concerns of men in recovery from substance addiction. Appetite. (2008) 50:33–42. 10.1016/j.appet.2007.05.006 17602790

[154] McDonald E Hedonic Mechanisms for Weight Changes in Medication Assisted Treatment for Opioid Addiction. University of Vermont (2017). Available online at: https://scholarworks.uvm.edu/cgi/viewcontent.cgi?article=1668&context=graddis

[155] Fenn J Laurent J Sigmon S. Increases in body mass index following initiation of methadone treatment. J Subst Abuse Treat. (2015) 51:59–63. 10.1016/j.jsat.2014.10.007 25441923 PMC4346498

[156] Peles E Schreiber S Sason A Adelson M. Risk factors for weight gain during methadone maintenance treatment. Subst Abus. (2016) 37:613–8. 10.1080/08897077.2016.1179705 27093441

[157] Housova J Wilczek H Haluzik M Kremen J Krizova J Haluzik M. Adipocyte-derived hormones in heroin addicts: the influence of methadone maintenance treatment. Physiol Res. (2005) 54:73–8. 15717844 10.33549/physiolres.930568

[158] Dursteler-MacFarland K Kowalewski R Bloch N Wiesbeck G Kraenzlin M Stohler R. Patients on injectable diacetylmorphine maintenance have low bone mass. Drug Alcohol Rev. (2011) 30:577–82. 10.1111/j.1465-3362.2010.00242.x 21355904

[159] Hardy R Fani N Jovanovic T Michopoulos V. Food addiction and substance addiction in women: Common clinical characteristics. Appetite. (2018) 120:367–73. 10.1016/j.appet.2017.09.026 28958901 PMC5680129

[160] Babrowski T Holbrook C Moss J Gottlieb L Valuckaite V Zaborin A. Pseudomonas aeruginosa virulence expression is directly activated by morphine and is capable of causing lethal gut-derived sepsis in mice during chronic morphine administration. Ann Surg. (2012) 255:386–93. 10.1097/SLA.0b013e3182331870 21989372 PMC3258463

[161] Meng J Yu H Ma J Wang J Banerjee S Charboneau R. Morphine induces bacterial translocation in mice by compromising intestinal barrier function in a TLR-dependent manner. PLoS ONE. (2013) 8:e54040. 10.1371/journal.pone.0054040 23349783 PMC3548814

[162] Banerjee S Sindberg G Wang F Meng J Sharma U Zhang L. Opioid-induced gut microbial disruption and bile dysregulation leads to gut barrier compromise and sustained systemic inflammation. Mucosal Immunol. (2016) 9:1418. 10.1038/mi.2016.9 26906406 PMC4996771

[163] Lee K Vuong HE Nusbaum DJ Hsiao EY Evans CJ Taylor AM. The gut microbiota mediates reward and sensory responses associated with regimen-selective morphine dependence. Neuropsychopharmacology. (2018) 43:2606–14. 10.1038/s41386-018-0211-9 30258112 PMC6224506

[164] Jadhav KS Peterson VL Halfon O Ahern G Fouhy F Stanton C. Gut microbiome correlates with altered striatal dopamine receptor expression in a model of compulsive alcohol seeking. Neuropharmacology. (2018) 141:249–59. 10.1016/j.neuropharm.2018.08.026 30172845

[165] Mansouri M Naghizadeh B Ghorbanzadeh B Alboghobeish S Amirgholami N Houshmand G. Venlafaxine prevents morphine antinociceptive tolerance: the role of neuroinflammation and the l-arginine-nitric oxide pathway. Exp Neurol. (2018) 303:134–41. 10.1016/j.expneurol.2018.02.009 29453978

[166] Yang C Fang X Zhan G Huang N Li S Bi J. Key role of gut microbiota in anhedonia-like phenotype in rodents with neuropathic pain. Transl Psychiatry. (2019) 9:57. 10.1038/s41398-019-0379-8 30705252 PMC6355832

[167] Richardson R Wiest K. A preliminary study examining nutritional risk factors, body mass index, and treatment retention in opioid-dependent patients. J Behav Health Serv Res. 42:401–8. 10.1007/s11414-013-9371-x 24091612

[168] Braveman P. Health disparities and health equity: concepts and measurement. Public Health. (2006) 27:167–94. 10.1146/annurev.publhealth.27.021405.102103 16533114

[169] Jeynes K Gibson E. The importance of nutrition in aiding recovery from substance use disorders: a review. Drug Alcohol Depend. (2017) 179:229–39. 10.1016/j.drugalcdep.2017.07.006 28806640

[170] Cunningham PM The use of sobriety nutritional therapy in the treatment of opioid addiction. J Addict Res Ther. (2016) 7:282 10.4172/2155-6105.1000282

[171] Waddington F Naunton M Kyle G Cooper G Nutritional intake of opioid replacement therapy patients in community pharmacies: A pilot study. Nutr Diet. (2015) 72:276–83. 10.1111/1747-0080.12192

[172] Leppert W. Emerging therapies for patients with symptoms of opioid-induced bowel dysfunction. Drug Des Devel Ther. (2015) 9:2215–31. 10.2147/DDDT.S32684 25931815 PMC4404965

[173] Barengolts E Green S Eisenberg Y Akbar A Reddivari B Layden B. Gut microbiota varies by opioid use, circulating leptin and oxytocin in African American men with diabetes and high burden of chronic disease. PLoS ONE. (2018) 13:e0194171. 10.1371/journal.pone.0194171 29596446 PMC5875756

[174] Camilleri M Lembo A Katzka DA. Opioids in gastroenterology: treating adverse effects and creating therapeutic benefits. Clin Gastroenterol Hepatol. (2017) 15:1338–49. 10.1016/j.cgh.2017.05.014 28529168 PMC5565678

[175] Poulsen JL Nilsson M Brock C Sandberg TH Krogh K ewes A. The Impact of Opioid Treatment on Regional Gastrointestinal Transit. J Neurogastroenterol Motil. (2016) 22:282–91. 10.5056/jnm15175 26811503 PMC4819867

[176] Nimmo W Heading R Wilson J Tothill P Prescott L. Inhibition of gastric emptying and drug absorption by narcotic analgesics. Br J Clin Pharmacol. (1975) 2:509–13. 10.1111/j.1365-2125.1975.tb00568.x 9953 PMC1402648

[177] Mora A Salazar M Pablo-Caeiro J Frost C Yadav Y DuPont H. Moderate to high use of opioid analgesics are associated with an increased risk of Clostridium difficile infection. Am J Med Sci. (2012) 343:277–80. 10.1097/MAJ.0b013e31822f42eb 21934595

[178] Acharya C Betrapally N Gillevet P Sterling R Akbarali H White M. Chronic opioid use is associated with altered gut microbiota and predicts readmissions in patients with cirrhosis. Aliment Pharmacol Ther. (2017) 45:319–31. 10.1111/apt.13858 27868217

[179] Kang M Mischel RA Bhave S Komla E Cho A Huang C. The effect of gut microbiome on tolerance to morphine mediated antinociception in mice. Sci Rep. (2017) 7:42658. 10.1038/srep42658 28211545 PMC5314392

[180] Wang F Temporal Modulation of Gut Microbiome and Metabolome by Morphine. University of Minnesota (2015). Available from: https://conservancy.umn.edu/handle/11299/177083

[181] Wang F Meng J Zhang L Johnson T Chen C Roy S. Morphine induces changes in the gut microbiome and metabolome in a morphine dependence model. Sci Rep. (2018) 8:3596. 10.1038/s41598-018-21915-8 29483538 PMC5827657

[182] Soto M Herzog C Pacheco JA Fujisaka S Bullock K Clish CB. Gut microbiota modulate neurobehavior through changes in brain insulin sensitivity and metabolism. Mol Psychiatry. (2018) 23:2287–301. 10.1038/s41380-018-0086-5 29910467 PMC6294739

[183] Cussotto S Clarke G Dinan TG Cryan JF. Psychotropics and the microbiome: a chamber of Secrets. Psychopharmacology. (2019) 236:1411–32. 10.1007/s00213-019-5185-8 30806744 PMC6598948

[184] Prescott SL Wegienka G Logan AC Katz DL. Dysbiotic drift and biopsychosocial medicine: how the microbiome links personal, public and planetary health. BioPsychoSoc Med. (2018) 12:7. 10.1186/s13030-018-0126-z 29743938 PMC5932796

[185] Vigo D Thornicroft G Atun R. Estimating the true global burden of mental illness. Lancet Psychiatry. (2016) 3:171–8. 10.1016/S2215-0366(15)00505-2 26851330

[186] Ali MM Dowd WN Classen T Mutter R Novak SP. Prescription drug monitoring programs, nonmedical use of prescription drugs, and heroin use: evidence from the national survey of drug use and health. Addict Behav. (2017) 69:65–77. 10.1016/j.addbeh.2017.01.011 28152391

[187] Pauly NJ Slavova S Delcher C Freeman PR Talbert J. Features of prescription drug monitoring programs associated with reduced rates of prescription opioid-related poisonings. Drug Alcohol Depend. (2018) 184:26–32. 10.1016/j.drugalcdep.2017.12.002 29402676 PMC5854200

[188] Glanz K Bishop DB. The role of behavioral science theory in development and implementation of public health interventions. Annu Rev Public Health. (2010) 31:399–418. 10.1146/annurev.publhealth.012809.103604 20070207

[189] McLeroy KR Bibeau D Steckler A Glanz K. An ecological perspective on health promotion programs. Health Educ Q. (1988) 15:351–77. 10.1177/109019818801500401 3068205

[190] Seymour RB Ring D Higgins T Hsu JR. Leading the way to solutions to the opioid epidemic: AOA critical issues. J Bone Joint Surg Am. (2017) 99:e113. 10.2106/JBJS.17.00066 29088045

[191] Smith RJ Kilaru AS Perrone J Paciotti B Barg FK Gadsden SM. How, why, and for whom do emergency medicine providers use prescription drug monitoring programs? Pain Med. (2015) 16:1122–31. 10.1111/pme.12700 25688454 PMC4478227

[192] García MC Dodek AB Kowalski T Fallon J Lee SH Iademarco MF. Declines in opioid prescribing after a private insurer policy change - Massachusetts, 2011-2015. MMWR Morb Mortal Wkly Rep. (2016) 65:1125–31. 10.15585/mmwr.mm6541a1 27764082

[193] Cochran G Field C Lawson K. Pharmacists who screen and discuss opioid misuse with patients. J Pharm Pract. (2015) 28:404–12. 10.1177/0897190014522064 24532819

[194] Penm J MacKinnon NJ Boone JM Ciaccia A McNamee C Winstanley EL. Strategies and policies to address the opioid epidemic: a case study of Ohio. J Am Pharm Assoc. (2017) 57:S148–53. 10.1016/j.japh.2017.01.001 28189539 PMC5497298

[195] Borrell-Carrió F Suchman AL Epstein RM. The biopsychosocial model 25 years later: principles, practice, and scientific inquiry. Ann Fam Med. (2004) 2:576–82. 10.1370/afm.245 15576544 PMC1466742

[196] Volkow ND Jones EB Einstein EB Wargo EM. Prevention and treatment of opioid misuse and addiction. JAMA Psychiatry. (2018) 76:208–16. 10.1001/jamapsychiatry.2018.3126 30516809

[197] Kaiser SK Prendergast K Ruter TJ Nutritional links to substance abuse recovery. J Addict Nurs. (2008) 19:125–9. 10.1080/10884600802305935

[198] Wiss DA Schellenberger M Prelip ML. Rapid assessment of nutrition services in Los Angeles substance use disorder treatment centers. J Commun Health. (2018) 44:1–7. 10.1007/s10900-018-0557-2 30030681

[199] Moore K Gray V Wiss D Parker E Hands-on nutrition and culinary intervention within a substance use disorder residential treatment facility. J Acad Nutr Diet. (2016) 116:A20 10.1016/j.jand.2016.06.058

[200] Wiss DA The role of nutrition in addiction recovery: what we know and what we don't. In: The Assessment and Treatment of Addiction: Best Practices and New Frontiers eds Danovitch I. Mooney L. J., St. Louis, MO: Elsevier (2019). p. 21–42. 10.1016/B978-0-323-54856-4.00002-X

[201] Wiss DA Schellenberger M Prelip ML. Registered dietitian nutritionists in substance use disorder treatment centers. J Acad Nutr Diet. (2018) 118:2217–21. 10.1016/j.jand.2017.08.113 29102421

[202] Schroeder RD Higgins GE. You are what you eat: the impact of nutrition on alcohol and drug use. Subst Use Misuse. (2017) 52:10–24. 10.1080/10826084.2016.1212603 27617497

[203] Chen D Liu Y He W Wang H Wang Z. Neurotransmitter-precursor-supplement intervention for detoxified heroin addicts. J Huazhong Univ Sci Technol Med Sci. (2012) 32:422–7. 10.1007/s11596-012-0073-z 22684569

[204] Sason A Adelson M Herzman-Harari S Peles E. Knowledge about nutrition, eating habits and weight reduction intervention among methadone maintenance treatment patients. J Subst Abuse Treat. (2018) 86:52–9. 10.1016/j.jsat.2017.12.008 29415851

[205] Grant L Haughton B Sachan D. Nutrition education is positively associated with substance abuse treatment program outcomes. J Am Diet Assoc. 104:604–10. 10.1016/j.jada.2004.01.008 15054346

[206] Barbadoro P Ponzio E Pertosa M Aliotta F D'Errico M Prospero E. The effects of educational intervention on nutritional behaviour in alcohol-dependent patients. Alcohol Alcohol. (2011) 46:77–9. 10.1093/alcalc/agq075 21097952

[207] Curd P Ohlmann K Bush H. Effectiveness of a voluntary nutrition education workshop in a state prison. J Correct Health Care. (2013) 19:144–50. 10.1177/1078345812474645 23481519

[208] Sandwell H Wheatley M Healthy eating advice as part of drug treatment in prisons. Prison Service J. (2009) 15–26.

[209] Cowan J Devine C. Process evaluation of an environmental and educational nutrition intervention in residential drug-treatment facilities. Public Health Nutr. (2012) 15:1159–67. 10.1017/S1368980012000572 22475412

[210] Cowan J Devine C. Diet and body composition outcomes of an environmental and educational intervention among men in treatment for substance addiction. J Nutr Educ Behav. (2013) 45:154–8. 10.1016/j.jneb.2011.10.011 22633178 PMC3430793

[211] Lindsay A Warren C Velasquez S Lu M. A gender-specific approach to improving substance abuse treatment for women: the healthy steps to freedom program. J Subst Abuse Treat. (2012) 43:61–9. 10.1016/j.jsat.2011.10.027 22154034

[212] Wall-Bassett E Robinson M Knight S “Moving Toward Healthy”: Insights into food choices of mothers in residential recovery. Glob Qual Nurs Res. (2016) 3:2333393616680902 10.1177/2333393616680902 28462350 PMC5342855

[213] Lu MC Halfon N. Racial and ethnic disparities in birth outcomes: a life-course perspective. Matern Child Health J. (2003) 7:13–30. 10.1023/A:1022537516969 12710797

[214] Lei M-K Beach SR Simons RL. Biological embedding of neighborhood disadvantage and collective efficacy: influences on chronic illness via accelerated cardiometabolic age. Dev Psychopathol. (2018) 30:1797–815. 10.1017/S0954579418000937 30106356 PMC6383366

[215] Glass TA McAtee MJ. Behavioral science at the crossroads in public health: extending horizons, envisioning the future. Soc Sci Med. (2006) 62:1650–71. 10.1016/j.socscimed.2005.08.044 16198467

[216] Lehrner A Yehuda R. Trauma across generations and paths to adaptation and resilience. Psychol Trauma. (2018) 10:22–9. 10.1037/tra0000302 29323523

[217] Umberson D Crosnoe R Reczek C. Social relationships and health behavior across life course. Annu Rev Sociol. (2010) 36:139–57. 10.1146/annurev-soc-070308-120011 21921974 PMC3171805

[218] Willson AE uey K Elder JH Cumulative advantage processes as mechanisms of inequality in life course health. Am J Sociol. (2007) 112:1886–924. 10.1086/512712

[219] Ben-Shlomo Y Kuh D. A life course approach to chronic disease epidemiology: conceptual models, empirical challenges and interdisciplinary perspectives. Int J Epidemiol. (2002) 31:285–93. 10.1093/intjepid/31.2.285 11980781

[220] Gordon EL Ariel-Donges AH Bauman V Merlo LJ. What is the evidence for “food addiction?” A systematic review. Nutrients. (2018) 10:477. 10.3390/nu10040477 29649120 PMC5946262

[221] Nabipour S Said AM Habil HM. Burden and nutritional deficiencies in opiate addiction- systematic review article. Iran J Public Health. (2014) 43:1022–32. 25927032 PMC4411899

[222] Jacka FN. Nutritional psychiatry: where to next? EBioMedicine. (2017) 17:24–9. 10.1016/j.ebiom.2017.02.020 28242200 PMC5360575

[223] Sarris J Logan AC Akbaraly TN Amminger PG Balanzá-Martínez V Freeman MP. Nutritional medicine as mainstream in psychiatry. Lancet Psychiatry. (2015) 2:271–4. 10.1016/S2215-0366(14)00051-0 26359904

[224] Grigoras CA Karanika S Velmahos E Alevizakos M Flokas M-E Kaspiris-Rousellis C. Correlation of opioid mortality with prescriptions and social determinants: a cross-sectional study of medicare enrollees. Drugs. (2018) 78:111–21. 10.1007/s40265-017-0846-6 29159797

[225] Dasgupta N Beletsky L Ciccarone D. Opioid crisis: no easy fix to its social and economic determinants. Am J Public Health. (2017) 108:e1–5. 10.2105/AJPH.2017.304187 29267060 PMC5846593

[226] Hemmingsson E. Early childhood obesity risk factors: socioeconomic adversity, family dysfunction, offspring distress, and junk food self-medication. Curr Obes Rep. (2018) 7:204–9. 10.1007/s13679-018-0310-2 29704182 PMC5958160

[227] Hemmingsson E Johansson K Reynisdottir S. Effects of childhood abuse on adult obesity: a systematic review and meta-analysis. Obes Rev. (2014) 15:882–93. 10.1111/obr.12216 25123205

[228] Smith RC The biopsychosocial revolution. J Gen Intern Med. (2002) 17:309–10. 10.1046/j.1525-1497.2002.20210.x

